# Describing behavioral aspects of annual lung cancer screening participation: Application of the cognitive-social health information processing model

**DOI:** 10.1080/28322134.2026.2690703

**Published:** 2026-06-22

**Authors:** Erin A. Hirsch, Kaitlyn Hoover, Stephen P. Malkoski, Betsy C. Risendal, Jamie L. Studts

**Affiliations:** a Department of Medicine, Division of Medical Oncology, University of Colorado School of Medicine, University of Colorado Anschutz Medical Center, Aurora, USA; b Cancer Prevention and Control Program, University of Colorado Cancer Center, University of Colorado Anschutz Medical Center, Aurora, USA; c Department of Medicine, University of Washington, WWAMI – Spokane, Spokane, USA; d Sound Critical Care, Sacred Heart Medical Center, Spokane, USA; e Department of Community and Behavioral Health, Colorado School of Public Health, University of Colorado Anschutz Medical Campus, Aurora, USA

**Keywords:** Lung cancer, lung cancer screening, health information processing, cognitive-social theory, health behavior

## Abstract

**Objective:**

Continued participation in yearly screening is necessary to maximize the effectiveness of lung cancer screening (LCS) with low-dose CT to detect early-stage disease. Determinants of LCS adherence are multi-faceted, rely heavily on factors related to health behavior, and need to be better understood. This exploratory study applied social-cognitive theory to characterize key demographic and clinical factors associated with LCS-specific health information processing related to annual adherence, with the goal of informing future hypothesis generation.

**Methods and Materials:**

This study employed a descriptive, cross-sectional survey of LCS-eligible individuals who had recently completed a low-dose CT and received results that recommended annual follow-up. Study participants completed a survey based on the Cognitive Social Health Information Processing model to describe health information processing and screening behaviors among individuals eligible for annual LCS.

**Results:**

The survey was completed by 143 LCS individuals with recommended annual follow-up, finding that this population has high self-efficacy about the LCS process, perceives the benefits as high, reports few barriers to continued annual screening, has low fatalism, but also reports suboptimal LCS-specific knowledge.

**Conclusion:**

These findings expand the limited literature addressing psychosocial and behavioral aspects of continued LCS participation and provide valuable information for future communication with LCS participants.

## Introduction

Lung cancer is a substantial public health burden in the United States. It is the second most common type of cancer diagnosed and the number one cause of cancer death among men and women, expected to account for approximately 11% of new cancer cases and 20% of all cancer deaths in 2025 [1]. Symptoms of lung cancer usually do not present until after the cancer has metastasized, directly influencing the high mortality [1]. When lung cancer is diagnosed at a localized, early stage, five-year survival exceeds 60%; however, it is only 7% when the disease is diagnosed at an advanced stage [2]. Currently, only 19% of cases are diagnosed early when more effective treatment options exist [2].

Lung cancer screening (LCS) with low-dose computed tomography (LDCT) is currently the only available method to effectively diagnose early-stage disease [3,4]. Findings from two large randomized controlled trials, the National Lung Screening Trial (NLST) [3] and the Dutch Belgian Lung Screening Trial (NELSON) [4], showed that LCS reduces lung cancer-specific mortality by at least 20% when participation in screening is consistent with recommended guidelines. LCS is recommended annually by the Centers for Medicare and Medicaid Services for individuals 50–77 years of age, with a minimum of 20 pack-years of tobacco exposure history, and who currently smoke or have quit within the past 15 years [5]. The US Preventive Services Task Force recommends screening up to 80 years of age [6].

The mortality reduction described in the NLST and NELSON studies was directly influenced by >90% adherence to follow-up rounds of screening [3,7]. Both trials diagnosed most early-stage cancers on follow-up rounds of screening compared to the initial LDCT scan [3,7], highlighting the importance of continued participation in recommended screening intervals. Importantly, the Tripartite Framework of Behavioral Adherence Elements to Lung Cancer Screening recognizes there are three types of adherence in the LCS process: baseline adherence (no-show rate to the baseline LDCT), interval adherence (follow-up of findings worrisome for lung cancer), and annual adherence (completion of a yearly LDCT for individuals that had previous negative result) [8]. The framework additionally emphasizes that each type of adherence may have different behavioral influences and need different interventions to support each element. *The research discussed in this paper focuses solely on annual adherence* as participation in yearly LCS has emerged as a core challenge for many LCS programs, with annual adherence rates reported to be 22% nationally and even lower in community and rural programs [9]. This low participation severely endangers the individual and population health efficacy and benefit of LCS.

While research has described sociodemographic [10–12] and system-level factors [10,13,14] associated with annual LCS that can be targeted for interventions, determinants of adherence are multi-faceted, rely heavily on factors related to health behavior, and warrant further investigation. Moreover, the LCS-eligible population experiences societal and self-stigma related to tobacco use histories [15] and often face disparities associated with social determinants of health [16], offering a complex challenge for effective intervention development and communication. Understanding behavioral aspects of LCS participation will inform intervention design and implementation, ultimately improving adherence rates and lung cancer survival.

To date, little is known about the behavioral aspects of LCS participation. Available research has focused on defining differences in health beliefs and psychosocial factors between LCS-eligible individuals who intend and do not intend to be screened [17–19]. To maximize LCS effectiveness, the literature needs a better understanding of how individuals eligible for annual LCS process screening-related health information and decide to continue obtaining yearly LDCT scans. We developed a survey instrument based on the Cognitive Social Health Information Processing model (C-SHIP) [20] to describe health information processing and screening behaviors among individuals eligible for annual LCS. C-SHIP was chosen to inform our survey because it combines constructs from the fields of health, cognitive, clinical, social, and behavioral psychology for robust measurement of how individuals process health information for health-protective behaviors, with the underlying premise that participation in health-protective behavior is an interaction between both cognitive–affective units and the environment through which these units interact [20]. C-SHIP was developed in the context of breast cancer screening and is comprised of five domains: (1) health-relevant encodings (strategies to encode for health, risks, and illness); (2) health beliefs and expectancies (beliefs and expectations related to outcomes and self-efficacy); (3) affects/emotions (emotions activated in health information processing); (4) health goals and values (desired and valued health outcomes); (5) self-regulatory competencies (knowledge and strategies for barriers) [20], that offer a comprehensive understanding of screening adherence behavior.

This exploratory study applied social-cognitive theory to characterize key demographic and clinical factors associated with LCS-specific health information processing related to annual adherence, with the goal of informing future hypothesis generation. Study findings will broaden the current understanding of behavioral attributes of participation to annual LCS, allowing this knowledge to encompass information on LCS-eligible individuals into targeted and/or tailored communication and intervention development.

## Methods and materials

This study employed a descriptive, cross-sectional survey of LCS-eligible individuals who had recently completed a LDCT and received results that recommended annual follow-up. Theoretical and conceptual frameworks used to guide this research and study methods have been described in detail elsewhere [21]. This research was approved by the Colorado Multiple Institutional Review Board (21–3881) and included waivers of informed consent to identify the study population. Additionally, documentation of informed consent was waived; respondents agreed to the contents of a short informational consent for inclusion in the study.

### Theoretical model and survey measures

Validated survey measures (described below) were selected to represent each C-SHIP domain after a review of how C-SHIP has been applied to breast, cervical, and prostate cancer research [20,22,23].

### Health-relevant encodings

*Lung cancer risk perception* was measured using the Lung Cancer Screening Health Belief Scales (LCSHBS) perceived risk subscale [17]. This subscale contains three questions answered on a 4-point Likert scale: strongly disagree (1), disagree (2), agree (3), and strongly agree (4) (score range 3 (low-risk perception) to 12 (high-risk perception)). *Functional well-being and health* were assessed using two questions adapted from the Health Information National Trends Cycle 5 Survey [24]. The first question (In general, would you say your health is …) had five answer options: Excellent, Very Good, Good, Fair, and Poor. The second question asked about respondents’ ability to take care of their own health (Overall, how confident are you about your ability to take good care of your health?) with answer options of completely confident, very confident, somewhat confident, a little confident, and not at all confident. The health worry/concern subscale from the Health Perceptions Questionnaire [25] was used to measure *health perception.* Four questions about worry and concern about health are answered on a 5-point Likert scale (definitely true (1), mostly true (2); I don’t know (3), mostly false (4), definitely false (5)). Summed scales can range from 4 (low concern) to 20 (high concern).

### Health beliefs and expectancies

The LCSHB self-efficacy subscale [17] was utilized to measure *LCS self-efficacy*. Questions were modified to ask about experiences with the recent LDCT (i.e. How confident were you …) and were answered on a 4-point Likert scale (not at all confident to very confident). The subscale included nine questions with an answer range of 9 (high confidence) to 36 (low confidence). *Beliefs about LCS for early detection* were measured using the Self-Regulatory Questionnaire for Lung Cancer Screening (SQR-LCS) behavioral response subscale [18], which contains three questions about the benefits and knowledge of lung cancer early detection. This subscale uses a 5-point Likert scale (strongly disagree (1), disagree (2), neither (3), agree (4), strongly agree (5)) for a range of scores from 3 (low beliefs) to 15 (high beliefs). *LCS knowledge* was evaluated using an existing 14-item instrument originally developed to assess knowledge before and after using a decision aid [26].

### Affects (emotions)

*Smoking-related stigma* was gauged using the five-question smoking subscale from the Cataldo Lung Cancer Stigma Scale [27]. For this scale, questions are answered on a 4-point Likert scale (strongly disagree to strongly agree) for a summed answer range of 5 (low stigma) to 20 (high stigma). The eleven-item revised Powe Fatalism Inventory [28] measured *lung cancer-related fatalism*. Fatalism questions were asked specifically about lung cancer and had Yes/No answer options. *Worry about a lung cancer diagnosis* was assessed from the SQR-LCS emotional representation subscale [18]. Three questions asked about worry, anxiety, and fear related to lung cancer were answered on a 5-point Likert scale (strongly disagree to strongly agree) with a scale range of 3 (low worry) to 15 (high worry).

### Health goals and values

*Benefits of LCS* were measured with the LCSHBS perceived benefits subscale [17] with six benefit-related questions using a 4-point Likert scale (strongly disagree to strongly agree). The summed scores range from 6 (low benefit) to 24 (high benefit). The Health Behavior Scale for Cancer Patients [29] measured *belief in a healthy lifestyle* among survey respondents. This scale poses nine questions on a 6-point scale (never, rare, sometimes, often, very often, always) about healthy lifestyle habits for a scale range of 9 (low healthy behaviors) to 54 (high healthy behavior). *Lung cancer illness coherence* and *consequences of a diagnosis* were gauged using the SQR-LCS illness coherence and consequences subscales [18]. Answers for both subscales are asked on a 5-point Likert scale (strongly disagree to strongly agree) for a summed scale range of 3 (low perceptions of illness/consequences) to 15 (high perception).

### Self-regulatory competencies

*Barriers to continued LCS* were measured with the 17-item LCSHBS perceived barriers subscale [17]. Questions were modified to query barriers to their recent LCS experience (i.e. I considered putting off having a lung scan …) and were answered on a 4-point Likert Scale (strongly agree to strongly disagree) to yield a measure score range from 17 (low barriers) to 68 (high barriers). The SQR-LCS personal control and treatment control subscales [18] were used to evaluate *control over a cancer diagnosis*. Each subscale contains three questions answered on a 5-point Likert scale (strongly disagree to strongly agree) for a range of 3 (low personal/treatment control) to 15 (high control). *Health literacy* was determined through the application of the BRIEF Health Literacy Scale [30], which asks four questions about difficulty and assistance needed for understanding and filling out health forms. Each question is answered on a 5-point Likert scale and summed for a scale score range of 4–20; scores of 4–12 are considered ‘limited’ health literacy, while scores of 13–16 and 17–20 are considered ‘marginal’ and ‘adequate’ literacy, respectively. The Subjective Numeracy Scale [31] measured *numeracy* with eight questions asking survey respondents to rate their comfort working with fractions and percentages and preferences for information to be presented using words or numbers. Questions were answered on 6-point Likert scales for an available scale range of 8 (low numeracy) to 48 (high numeracy). *Approach to healthcare and seeking information* was gauged using the Medical Maximizer-Minimizer 1 Scale [32], a single-item measure that asks about preferences for pursuing healthcare. This scale is answered on a 6-point Likert scale (strongly lean toward waiting and seeing (1), lean toward waiting and seeing (2), somewhat lean toward waiting and seeing (3), somewhat lean toward taking action (4), lean toward taking action (5), strongly lean toward taking action (6)).

### Respondent characteristics

The questionnaire also included sociodemographic questions (age, sex at birth, current gender, employment status, type of health insurance, marital status, education, race, ethnicity, and household income), medical history questions (family history of lung cancer in parents, siblings, or children and previous diagnosis of cancer, diabetes or high blood sugar, high blood pressure or hypertension, heart conditions including heart attack, angina, or congestive heart failure, chronic lung diseases defined as asthma, emphysema, or chronic bronchitis, and depression/anxiety), and smoking history (age when first started to smoke cigarettes, current cigarette smoking status, age when last smoked (for those who formerly smoked), and number of cigarettes smoked per day on average). Additionally, respondents were asked whether a clinician had recommended they have an LCS LDCT, whether a clinician told them LCS should be done yearly, and their intent to adhere to yearly LCS was assessed on a 5-point Likert scale (strongly disagree to strongly agree) with the question ‘I plan to have another low dose CT for lung cancer screening in about a year from now’.

### Sample size

The accrual goal for this study was a minimum of 125 completed surveys. Assuming this sample size and using a two-sample proportion test, the study had 80% power to detect a minimum difference of 20% for a chi-squared test with 1 degree of freedom or 28% for a chi-squared test with 2 degrees of freedom between C-SHIP constructs and survey respondent characteristics at an alpha level of 0.05. We used the Tailored Design Method [33] to maximize response rates; specifically, non-respondents were contacted multiple times and provided compensation for survey completion.

### Study eligibility, data collection, and survey distribution

Participants were recruited from three lung cancer screening programs across Colorado: the University of Colorado Hospital (UCH) in Aurora, CO, UCHealth North in Fort Collins, CO, and UCHealth South in Colorado Springs, CO. Eligible individuals were identified from LCS program clinical databases. Surveys were sent to all individuals who met CMS or USPSTF guidelines and had undergone a screening-specific LDCT, with results recommending annual follow-up (LungRADs 1 or 2) within 1–2 weeks prior to study invitation. Individuals who were ineligible for clinically recommended LCS or had CT results recommending shorter-term follow-up (LungRADs 3 or 4) were excluded from study invitation.

Eligible individuals with an email address available in the electronic health record were emailed a study invitation that included a link to a web-based REDCap survey (Research Electronic Data Capture; Vanderbilt University; electronic data capture hosted at the University of Colorado Anschutz Medical Campus) [34,35]. The initial invitation was followed by two reminder emails, and a final mailed letter and paper survey was sent to the individual’s postal address if a response was not received from the web-based survey. Eligible individuals with only a postal address in the electronic health record were mailed a paper survey and two reminder letters. Respondents received a $40 gift card upon completion.

### Statistical analysis

Descriptive statistics and univariate differences between the sampling population and survey respondents were assessed using Fisher’s Exact or t-tests for categorical and continuous variables, respectively. Questions within survey scales were summed, and the distribution for each measure was assessed using quantile-quantile plots with normality checked with the Shapiro–Wilk test. Single-item scales were categorized. Responses with missing data for Likert-type scales were only excluded from that measure’s analysis. Missing data from scales with Yes/No answer options (knowledge and fatalism scales) were recoded to a ‘No’ answer. Internal consistency and scale reliability for each Likert-type measure were assessed by calculating Cronbach’s alpha [36]. Univariate differences between respondent characteristics and C-SHIP measures were determined by t-test or the Wilcoxon Rank Sum test for parametric and non-parametric continuous measures, respectively, and with a chi-square test for categorical variables. Data was analyzed with SAS/STAT, v9.4 (SAS Institute Inc., Cary, NC, USA).

## Results

Surveys were sent to 287 LCS participants between July and December 2022; 143 responses were returned (93 from UCH, 38 from UCHealth North, and 12 from UCHealth South), yielding a 50% response rate. Characteristics of the full sample and subsample of survey respondents are shown in Table 1. Survey respondents were significantly more likely to have formerly smoked cigarettes (vs. currently smoking cigarettes) than the sampling population (69% versus 57%, *p* = 0.02). Additional sociodemographic and medical characteristics of survey respondents are presented in Table 2: 19% of respondents had a minimum of a high school education, 52% had an annual income of <$75,000, and 25% reported a family history of lung cancer in a first-degree relative (sibling, parent, or child). Nearly all respondents reported they plan on remaining adherent to annual screening (92%).

### Health relevant encodings (Table 3)

#### Perceived lung cancer risk

Survey respondents reported a median perceived lung cancer risk score of 6.0 [interquartile range (IQR) 6.0–8.0; range = 3.0 (low perceived risk) to 12.0 (high perceived risk)]. Individuals who currently smoke cigarettes reported a higher perceived risk when compared to individuals who formerly smoked, with median scores of 6.0 (IQR 6.0–9.0) and 6.0 (IQR 6.0–7.0), respectively (*p* = 0.008). Likewise, survey respondents with a family history of lung cancer had a significantly higher perceived risk of lung cancer of 7.0 (IQR 6.0–9.0) than individuals without a family history of 6.0 (IQR 6.0–7.0) (*p* = 0.001).

#### Self-rating of current health

Overall, 71 (50%) of respondents rated their health as good or excellent. Individuals with private insurance (*p* = 0.02), a college education (*p* = 0.05), an annual income >$75,000 (*p* = 0.02), no prior history of chronic lung disease (*p* = 0.002), or previous anxiety or depression (*p* = 0.009) were more likely to rate their health as good or excellent.

#### Self-rating of ability to take care of own health

Of survey respondents, 82 (57%) of respondents reported they were completely or very confident in their ability to take care of their health. Males had higher confidence about caring for their health than females (68% versus 32%, *p* = 0.005). Individuals who formerly smoked cigarettes (*p* = 0.006) and those without a history of anxiety or depression (*p* = 0.0001) also reported significantly higher confidence levels in their ability to take care of their health.

#### Health perception

Respondents reported moderate levels of health worry or concern [median score of 11.0 (IQR 9.0–12.0); range = 4.0 (low worry) to 18.0 (high worry)]. Survey respondents who were non-Hispanic White reported significantly higher worry about their health than respondents who self-identified as another race or ethnicity [median scores (IQR) of 11.0 (9.0–12.5) vs. 10.0 (8.0–10.0), *p* = 0.007].

### Health beliefs and expectancies (Table 4)

#### LCS self-efficacy

Levels of LCS self-efficacy were high among survey respondents [median score 35.0 (IQR 32.0–36.0); range = 12.0 (low self-efficacy) to 36.0 (high self-efficacy)]. Respondents with an income >$75,000 reported higher levels of self-efficacy compared to individuals with incomes <$75,000, with median summed scores of 36.0 (IQR 34.0–36.0) and 34.0 (IQR 30.0–36.0), respectively (*p* = 0.03). Individuals reporting a diagnosis of diabetes (*p* = 0.02) or heart condition (*p* = 0.03) also reported higher levels of LCS self-efficacy.

#### Beliefs about LCS for early detection

The median score for belief in LCS early detection was 14.0 (IQR 11.0–15.0) with a range of 3.0 (low positive perception) to 15.0 (high positive perception), indicating high levels of positive perceptions of LCS early detection. No significant differences in beliefs about early detection were found between respondent attributes.

#### LCS-specific knowledge

Levels of LCS knowledge were low among survey participants, with a median number of correctly answered questions of 4.0 (IQR 2.0–6.0), with a range of 0–12 correctly answered questions. Females answered significantly more questions correctly than males (*p* < 0.0001), with the median number of correct answers 6.0 (IQR 4.0–7.5) for females compared to 3.0 (IQR 2.0–5.0) for men. Survey respondents who reported their race and ethnicity to be Non-Hispanic White also correctly answered more knowledge questions than respondents of other races and ethnicities [median correct answers 4.0 (IQR 3.0–6.0) and 3.0 (IQR 2.0–4.0), respectively] (*p* = 0.03). Responses for each individual knowledge question are included in Supplemental Table 2.

### Affects (Emotions) (Table 5)

#### Smoking-related stigma

Survey respondents reported a median stigma score of 14.0 (IQR 12.0–14.0) with a range of 5.0 (low stigma) to 19.0 (high stigma), indicating moderate levels of stigma. Individuals with a high school education reported lower levels of stigma (median score of 12.5 (IQR 11.5–14.0)) compared to individuals with a college education (median score of 14.0 (IQR 13.0–14.0) (*p* = 0.02)).

#### Lung cancer-related fatalism

Overall, there were low levels of fatalism among survey respondents, with a median score of 1.0 (IQR 0.0–2.0) and a range of 0.0 (low fatalism) to 11.0 (high fatalism). However, survey respondents with a high school education (*p* = 0.01) and those with a family history of lung cancer (*p* = 0.01) reported significantly higher levels of fatalism than individuals with a college education or no family history.

#### Perceived worry about a lung cancer diagnosis

The median summed score for worry about a lung cancer diagnosis was 11.0 (IQR 9.0–12.0), with a range of 3.0 (low worry) to 15.0 (high worry), indicating moderate levels of perceived worry. Individuals who were 50–64 years of age (*p* = 0.01), had a family history of lung cancer (*p* = 0.05), and those with a history of anxiety or depression (*p* = 0.006) reported higher worry.

### Health goals and values (Table 6)

### Perceived LCS benefits

Perceived LCS benefits were high among survey respondents; the median score was 22.0 (IQR 18.0–24.0) with a range of 14.0 (low benefit) to 24.0 (high benefit). No significant differences in perceived benefits were found between respondent attributes.

#### Belief in healthy lifestyle

The mean healthy behavior score was 37.4, with a standard deviation of 6.7, indicating moderate levels of healthy behaviors, with a range of 21.0 (low levels of healthy behavior) to 52.0 (high levels of healthy behavior). Individuals with a college degree were more likely to report higher levels of healthy behaviors than individuals with a high school education (mean scores of 38.1 (±6.8) and 34.5 (±5.6), respectively; *p* = 0.01).

#### Illness coherence

Survey respondents had a summed median illness coherence score of 8.0 (IQR 6.0–10.0) with a range of 3.0 (low coherence) to 15.0 (high coherence), suggesting moderate levels of illness coherence. Survey respondents with a history of high blood pressure had lower illness coherence than respondents without a high blood pressure diagnosis (*p* = 0.03), with median scores of 8.0 (IQR 6.0–10.0) for individuals with high blood pressure compared to 9.0 (IQR 7.0–11.0) without.

#### Consequence of a lung cancer diagnosis

Levels of perceived consequences of a lung cancer diagnosis for an individual and family were high among survey respondents, with a median score of 14.0 (IQR 12.0–15.0), range 3.0 (low consequences) to 15.0 (high consequences). Respondents with private insurance reported significantly higher levels of perceived consequences than respondents with government-issued insurance, with median scores of 15.0 (IQR 13.0–15.0) and 14.0 (IQR 12.0–15.0), respectively (*p* = 0.05).

### Self-regulatory competencies (Table 7)

#### Perceived LCS barriers

Overall, there were low levels of perceived barriers to LCS among respondents, with a median score of 24.5 (IQR 17.0–32.0) and a range of scores from 17.0 (low barriers) to 46.0 (high barriers). Individuals with a previous chronic lung disease diagnosis reported more barriers than individuals without a chronic lung disease [median scores of 28.0 (IQR 19.0–34.0) and 20.0 (IQR 17.0–28.0), *p* = 0.005].

#### Personal control

Survey respondents had a median personal control score of 12.0 (IQR 11.0–13.0), suggesting high levels of personal control over a cancer diagnosis, with a range of scores from 3.0 (low perceived personal control) to 15.0 (high perceived personal control). Individuals that were 50–64 years of age reported higher personal control compared to individuals 65–77 years of age (median scores 12.0 (IQR 11.0–14.0) versus 12.0 (IQR 10.0–13.0), *p* = 0.04).

#### Treatment control

The overall median summed treatment control score was 10.0 (IQR 9.0–12.0), indicating moderate levels of perceived treatment control. Scores ranged from 3.0 (low perceived treatment control) to 15.0 (high perceived treatment control). Survey respondents with a family history of lung cancer had lower levels of perceived treatment control than respondents without a family history, with median scores of 10.0 (IQR 9.0–11.0) and 11.0 (IQR 10.0–12.0), respectively (*p* = 0.01).

#### Health literacy

Overall, survey respondents reported ‘adequate’ literacy with a median score of 18.0 (IQR 15.0–20.0) and a range of literacy scores from 5.0 (‘limited’ literacy) to 20.0 (‘adequate’ literacy). Respondents with a college education (*p* = 0.001), an income >$75,000 (*p* = 0.002), and without a previous heart condition diagnosis (*p* = 0.003) had median health literacy scores in the ‘adequate’ range. In contrast, individuals with a high school education, income <$75,000, and a heart condition diagnosis had median scores in the ‘marginal’ health literacy range.

#### Numeracy

The median summed numeracy score was 38.5 (IQR 30.0–44.0), with a range of scores from 8.0 (low numeracy) to 48.0 (high numeracy). Similar to literacy, individuals with a college education (*p* < 0.0001), an income >$75,000 (*p* < 0.0001), and without a previous heart condition diagnosis (*p* = 0.03) had significantly higher median numeracy scores.

#### Medical minimizer or maximizer

A slight majority of survey respondents (53%) rated themselves as medical ‘minimizers’. Respondents with a college education reported being more likely to have a ‘wait and see’ approach to healthcare than respondents with a high school education (25% vs. 75%, respectively, *p* = 0.03). Individuals without a history of anxiety or depression were also more likely to be healthcare minimizers than individuals with anxiety or depression (*p* = 0.02).

## Discussion

Yearly participation in LCS is vital to diagnose early-stage disease, increase 5-year survival, and ultimately decrease the individual and societal burden of lung cancer. Since the LCS-eligible population often faces increased adverse social drivers of health, mistrust of the healthcare system, and stigma related to having a history of cigarette smoking [15,37,38], a thorough understanding of behavioral factors is needed to guide intervention and communication development to help increase LCS participation. Adhering to annual screening is a complex behavioral decision comprised of individual psychological and environmental influences that need to be better understood by clinicians and researchers. We developed a comprehensive survey based on the robust C-SHIP model [20], allowing for the measurement and evaluation of several constructs that influence LCS health decisions. To the best of our knowledge, this is the first study to describe health information processing factors specific to annual screening participation, providing valuable information that can be used for future intervention and communication development.

### Health-relevant encodings

The health-relevant encodings domain was operationalized to evaluate LCS participation through risk perception and self-rating of health and functional well-being. Perceived lung cancer risk was consistent with previous applications of the LCSHBS, with a median score of 6.0 (moderate risk perception) also previously reported in LCS-eligible populations that had been screened or intended to be screened in the United States, Spain, and China [17,39,40]. Also consistent with previous research, we found that respondents who formerly smoked and had a family history of lung cancer had a higher perceived risk of developing lung cancer [41–43]. High-risk perception has been associated with participation in colorectal cancer screening and is likely a driving factor for continued adherence [44], but could work differently in the LCS context when combined with other psychosocial constructs such as fatalism or stigma driven by prominent fear-based tobacco control and public health messaging.

The majority of survey respondents rated their health as good or excellent and had high confidence in their ability to take care of their health. This is in contrast to previous research that found individuals with poorer status were more likely to report being up-to-date with LCS than individuals with good health status from analysis of data from the Behavioral Risk Factor Surveillance System [45]. It is possible that this survey attracted healthier people who are more likely to receive preventive care, including cancer screenings. Nonetheless, several sociodemographic and medical characteristics emerged with lower-rated health that are consistent with indicators of low SES, including individuals with government-sponsored insurance, a high school education, income <$75,000, and those with chronic lung disease or depression/anxiety diagnosis [16,46–48]. Furthermore, 57% of this sample were very or completely confident in their ability to take care of their health, with men, those who formerly smoked, and individuals without anxiety/depression reporting significantly higher confidence than women, individuals who currently smoke, or have an anxiety/depression history. These observations are consistent with prior research that has found women have lower self-efficacy for maintaining physical activity [49], while individuals who formerly smoke are more likely to participate in preventive care [50], and self-care can be a burden for individuals with depression [51].

### Health beliefs and expectancies

The survey measured LCS self-efficacy, beliefs about early lung cancer detection, and knowledge to evaluate the health beliefs and expectancies domain among LCS-eligible individuals. Self-efficacy determined by the LCSHBS was overall higher among survey respondents (median score 35.0 (range of 9.0–36.0)) than previously reported among individuals who had been screened or intended to be screened [17,39,40], possibly suggesting that high levels of self-efficacy may be related to increased adherence as eligible individuals gain experience with the screening process. Among survey respondents, lower-income individuals reported lower self-efficacy, and respondents with a history of diabetes and heart conditions reported higher LCS self-efficacy, potentially due to more experience navigating the healthcare system [52]. Behavioral response for beliefs in early detection was high among the survey sample and aligned with the previous application of the SQR-LCS among individuals who intended to be screened for lung cancer [18,19]. Similar to previous research, survey respondents had limited LCS knowledge [26,53], with a median of four (IQR 2.0–6.0) correctly answered questions (out of 14). Our observation that women and Non-Hispanic White respondents answered significantly more questions correctly might be attributed to their higher use and more confidence in using the internet to seek out health information [54,55].

### Affects (Emotions)

Affective states measured in this survey included smoking-related stigma, lung cancer-related fatalism, and perceived worry about a lung cancer diagnosis. The overall level of stigma in the survey population and lower levels of stigma among respondents with a high school education are in line with earlier reports of stigma measured with the Cataldo Lung Cancer Stigma Scale [56], potentially related to differences in acceptability of smoking among high and low SES [57]. Interestingly, there were low levels of fatalism among the survey population. Increased fatalism has been previously associated with avoidance of LCS [58], potentially indicating that low levels of fatalism are important in annual LCS. Higher levels of fatalism for individuals with a high school education and a family history of lung cancer have commonly been associated as a barrier to cancer screening [59–62]. Last, our survey sample had high positive emotional representation for worry about a lung cancer diagnosis; this rating contrasts prior validation of the SQR-LCS that reported positive emotional representation was associated with less screening intention [18].

### Health goals and values

We operationalized the C-SHIP health goals and values domain with scales to measure perceived LCS benefits, respondent belief in a healthy lifestyle, illness coherence, and consequences of a lung cancer diagnosis. Levels of perceived benefits were higher in our survey population (median 22.0 (IQR 18.0–24.0)) than reported with prior usage of the LCSHBS in LCS-eligible populations that had or intended to be screened [17,39,40]. Placing high value on the benefits of LCS may be highly important for continued LCS participation. Single-item questions asking about the frequency of healthy behaviors were similar to the mean scores reported in German breast cancer patients [28]. Consistent with other research, we observed that survey respondents with a high school education had fewer healthy behaviors than college-educated individuals [63]. Illness coherence in our population was similar to previous use of the scale in English individuals who intended to be screened [18,19]; however, we observed much higher levels of perceived consequences of a lung cancer diagnosis. One possible explanation is that our survey sample perceives the seriousness of a cancer diagnosis as higher after undergoing shared decision-making to discuss screening-related risks and benefits, a requirement for Medicare coverage of LCS in the United States [5].

### Self-regulatory competencies

The survey evaluated the self-regulatory competencies domain by measuring LCS perceived barriers, approach to health care, perceived personal and treatment control of a cancer diagnosis, and subjective health literacy and numeracy. Barriers to continued LCS were low among survey respondents (median score 24.5 (IQR 17–32)) and lower than previous LCSHBS barriers rated by individuals who had been screened or intended to be screened [17,39,40], potentially indicating that few barriers are another important factor for annual LCS participation. Interestingly, respondents with a previous chronic lung diagnosis reported higher barriers, consistent with high, multi-level barriers related to pulmonary rehabilitation [64,65]. Ratings for both personal and treatment control were similar for values reported for individuals who intended to undergo LCS in validation of the SRQ-LCS [18]. Less personal control among older ages and higher perceived effectiveness of treatment among Black, Asian, and mixed/multiple race individuals are consistent with our observed findings [19].

While our overall survey population had ‘adequate’ health literacy and high subjective numeracy, the observation that respondents with a high school education and lower income reported lower scores is well documented in the disease prevention literature [66–68]. Survey respondents with a diagnosed heart condition also had lower literacy and numeracy, which is likely explained by these individuals having more individuals with an income of <$75,000 and a high school education than those with other medical diagnoses. A slight majority (53%) of respondents to our survey reported they were healthcare minimizers, preferring a ‘wait and see’ approach to seeking out healthcare. Previous prostate cancer screening research that used the 10-item Medical Maximizing-Minimizing Scale [69] (a longer version of the 1-item Medical Maximizing-Minimizing scale we used) found that healthcare maximizers usually have a higher intent to screen [70] and, similar to our study, reported that individuals with less education were more likely to be healthcare minimizers [71].

### Implications for LCS communication and intervention development

This descriptive analysis aimed to examine how individuals eligible for annual LCS represent and process health information to guide future communication and intervention development. Based on principles of user-centered design to encompass information about the target population into the design of interventions, we utilized C-SHIP to consider several factors that influence health behavior, including what eligible individuals think, feel, and manage expectations related to LCS [72]. Health communication interventions designed based on theory have resulted in greater uptake of health-protective behaviors than communication designed without theory [73]. This study found that LCS individuals with recommended annual follow-up have high self-efficacy about the LCS process, perceive the benefits as high, report few barriers to continued annual screening, and have low LCS-specific knowledge and fatalism. Additionally, we found several statistically significant differences between survey measures and respondent sociodemographic and medical characteristics related to differences in how LCS health information is processed (Table 8), offering potential opportunities for communication and intervention targeting and/or tailoring. It is imperative to point out that these opportunities are specific for *annual LCS participation*. Consistent with the Tripartite Framework of Behavioral Adherence Elements of Lung Cancer Screening [8], behavioral factors that influence baseline and interval adherence may be different than elements related to annual adherence; additional research is needed to further understand behavioral components across the entire screening process.

Another crucial observation was that some survey respondents had limited health literacy and numeracy, highlighting the importance of developing communication materials in plain language at an appropriate reading level [74]. Furthermore, since low health literacy can be an indicator of social determinants of health [66], future LCS adherence interventions should consider the impact of health literacy and other social determinants of health. Cancer screening interventions that consider social determinants of health have been shown to increase screening rates for breast, cervical, colorectal, and lung cancer screening [75].

### Implications for future LCS participation research

The use of the C-SHIP model provides a robust framework to measure several hypothesized behavioral influences of LCS annual adherence participation. Although our study is descriptive and not intended to define factors associated with screening participation, we observed several differences between our results and previously reported social and health belief factors related to LCS uptake and intent to be screened. Specifically, LCS-eligible individuals who have completed at least one LDCT and remain eligible for annual screening reported higher self-efficacy, lower fatalism, more worry about a lung cancer diagnosis, perceived high benefit, high perceived consequences of a cancer diagnosis, and low LCS barriers compared to individuals who intended to be screened [17–19,39,40]. These observed differences may be important distinctions between individuals contemplating participation in LCS for the first time compared to those needing to adhere to annual screening. More research is needed in larger, adequately powered studies to confirm the true relationship and influence of mediators and/or moderators of these factors on continued participation in yearly LCS.

### Limitations

This study does have several limitations that must be acknowledged. First, our survey population represented fewer individuals who currently smoke than our sampling population, potentially introducing bias into our results. Specifically, LCS health information processing beliefs may differ between individuals who currently and formerly smoked. Second, our survey population was composed entirely of individuals from Colorado, an area that is racially less diverse with lower smoking rates than other areas of the United States [76], potentially limiting the generalizability of our results to other LCS-eligible individuals. Last, we could not assess health information processing in some populations that stand to benefit significantly from LCS, such as the LGBTQ population, individuals who suffer from mental and substance abuse disorders, and populations that have structural barriers to LCS participation. Future research is needed to explore health information processing in these populations to close equity gaps and eliminate health disparities.

## Conclusions

Despite these limitations, this study expands the limited body of knowledge on psychosocial and behavioral aspects of continued LCS participation, demonstrates possible differences in health information processing based on sociodemographic and medical characteristics, and provides valuable information for future communication with LCS participants. Understanding health information processing among these populations and differences based on individual characteristics will lead to effective and efficient communication and interventions that can be targeted or tailored to achieve optimal LCS participation and ultimately improve lung cancer survivorship.

## Supplementary Material

Supp 1

Supplemental data for this article can be accessed online at https://doi.org/10.1080/28322134.2026.2690703.

## Figures and Tables

**Table 1.** Characteristics of sampling population and survey respondents.

	Sampling population n = 287	Survey respondents n = 143	p-Value

Sex*			
Male	162 (56.4)	82 (57.8)	0.80
Female	125 (43.6)	60 (42.2) Missing n = 1	
Race/ethnicity			
Non-Hispanic White	250 (87.1)	125 (90.6)	0.30
Other race/ethnicity	37 (12.9)	13 (9.4) Missing n = 5	
Age (mean (±SD))	66.0(±6.0)	65.5(±6.2)	0.41
Age (range)	50–77	50–77 Missing n = 5	
Smoking status			
Current	123 (43.3)	44 (31.0)	0.02
Former	161 (56.7) Missing n = 3	98 (69.0) Missing n = 1	

Data are presented as count (%) or mean (± standard deviation).

* Biological sex at birth. While the survey asked about sex at birth and current gender identity, data were analyzed as sex at birth, as only one survey respondent indicated they were transgender.

**Table 2.** Sociodemographic and medical characteristics of the survey population.

	Survey population n = 143

Insurance (missing n = 1)	
Private	44 (31.0)
Government-sponsored (Medicare, Medicaid, TriCare, VA)	98 (69.0)
Education (missing n = 3)	
High School	27 (19.3)
College	133 (80.7)
Income (missing n = 8)	
Less than $75,000	70 (51.8)
More than $75,000	65 (48.2)
Current working status*	
Working	46 (32.2)
Not working (unemployed, disabled, retired)	97 (67.8)
Current marital status*	
Married	83 (58.0)
Not married (divorced, widowed, separated, co-inhabiting)	60 (42.0)
Family history of lung cancer	
Yes	36 (25.2)
No	107 (74.8)
Previous cancer diagnosis (missing n = 2)	
Yes	29 (20.6)
No	112 (79.4)
Previous diabetes diagnosis (missing n = 1)	
Yes	38 (26.8)
No	104 (73.2)
Previous high blood pressure diagnosis	
Yes	84 (58.7)
No	59 (41.3)
Previous heart condition diagnosis (missing n = 1)	
Yes (heart attack, angina, congestive heart failure)	24 (16.9)
No	118 (83.1)
Previous chronic lung disease diagnosis (missing n = 1)	
Yes (asthma, emphysema, chronic bronchitis)	69 (48.6)
No	73 (51.4)
Previous depression or anxiety diagnosis (missing n = 2)	
Yes	45 (31.9)
No	96 (68.1)
Clinician recommended LCS (missing n = 2)	
Yes	129 (91.5)
No	12 (8.5)
Clinician counseled that LCS should be completed yearly (missing n = 1)	
Yes	121 (85.2)
No	21 (14.8)
Respondents intend to adhere to yearly LCS (missing n = 2)	
Agree/strongly agree	129 (91.5)
Disagree/strongly disagree	12 (8.5)

Data are presented as counts (%).

* Current working and marital status are not included in additional analyses as no significant differences existed for any lung cancer screening measures.

**Table 3.** Health-relevant encodings domain results.

	Lung cancer risk = 138*	*p*-Value	Self-health rating n = 143		*p*-Value	Ability to take care of health = 143		*p*-Value	Health perceptions N = 142^†^	*p*-Value
			Good/Excellent n = 101	Fair/Poor n = 42		Higher confidence N = 82	Lower confidence N = 61			

Cronbach alpha	0.90		–	–		–	–		0.67	
Median overall score (IQR)	6.0(6.0–8.0)		–	–		–	–		11.0(9.0–12.0)	
Scale answer range	3.0–12.0		–	–		–	–		4.0–18.0	
Sex										
Male	6.0(6.0–8.0)	0.61	61(61.0)	21(50.0)	0.23	55(67.9)	27(44.3)	**0.005**	11.0(10.0–12.0)	0.78
Female	6.0(6.0–7.0)		39(39.0)	21(50.0)		26(32.1)	34(55.7)		11.0(9.0–13.0)	
Race/Ethnicity										
Non-Hispanic White	6.0(6.0–7.5)	0.18	89(91.8)	36(87.8)	0.53	72(91.1)	53(89.8)	0.79	11.0(9.0–12.5)	**0.007**
Other race/ethnicity	7.0(6.0–9.0)		8(8.5)	5(12.2)		7(8.9)	6(10.8)		10.0(8.0–10.0)	
Age										
50–64	6.0(6.0–8.0)	0.93	41(42.3)	18(43.9)	0.86	32(40.0)	27(56.6)	0.44	11.0(9.0–12.0)	0.17
65–77	6.0(6.0–8.0)		56(57.7)	23(56.1)		48(60.0)	31(53.4)		11.0(9.0–13.0)	
Smoking status										
Current	6.0(6.0–9.0)	**0.008**	31(30.7)	13(31.7)	0.91	18(21.9)	26(43.3)	**0.006**	10.0(9.0–12.0)	0.33
Former	6.0(6.0–7.0)		70(69.3)	28(68.3)		64(78.1)	34(56.7)		11.0(9.0–13.0)	
Insurance										
Private	6.0(6.0–8.0)	0.53	37(37.0)	7(16.7)	**0.02**	27(33.3)	17(27.9)	0.49	11.0(9.0–12.0)	0.82
Government	6.0(6.0–8.0)		63(63.0)	35(83.3)		54(66.7)	44(72.1)		11.0(9.0–12.0)	
Education										
High School	6.0(6.0–8.0)	0.70	15(15.2)	12(29.3)	**0.05**	16(20.0)	11(18.3)	0.80	11.0(9.0–13.0)	0.88
College	6.0(6.0–7.5)		84(84.8)	29(70.7)		64(80.0)	49(81.7)		11.0(9.0–12.0)	
Income										
<$75,000	6.0(6.0–8.0)	0.18	43(45.3)	27(67.5)	**0.02**	37(47.4)	33(57.9)	0.23	11.0(9.0–13.0)	0.35
> $75,000	6.0(6.0–7.0)		52(54.7)	13(32.5)		41(52.6)	24(42.1)		11.0(10.0–12.0)	
Family history of lung cancer										
Yes	7.0(6.0–9.0)	**0.001**	23(22.8)	13(30.9)	0.31	19(23.2)	17(27.9)	0.52	10.0(9.0–12.0)	0.30
No	6.0(6.0–7.0)		78(77.2)	29(69.1)		63(76.8)	44(72.1)		11.0(10.0–12.0)	
Previous cancer diagnosis										
Yes	6.0(6.0–7.5)	0.64	20(20.0)	9(21.9)	0.79	14(17.3)	15(25.0)	0.26	11.0(10.0–12.0)	0.43
No	6.0(6.0–8.0)		80(80.0)	32(78.1)		67(82.7)	45(75.0)		11.0(9.0–13.0)	
Previous diabetes diagnosis										
Yes	6.0(6.0–7.0)	0.30	23(23.0)	15(35.7)	0.12	22(27.2)	16(26.2)	0.90	11.0(10.0–13.0)	0.53
No	6.0(6.0–8.0)		77(77.0)	27(64.3)		59(72.8)	45(73.8)		11.0(9.0–12.0)	
Previous HBP diagnosis										
Yes	6.0(6.0–7.0)	0.37	55(54.5)	29(69.1)	0.11	46(56.1)	38(62.3)	0.46	11.0(9.5–12.0)	0.96
No	6.0(6.0–8.0)		46(45.5)	13(30.9)		36(43.9)	23(37.7)		11.0(9.0–12.0)	
Previous heart condition diagnosis										
Yes	6.0(6.0–8.0)	0.93	13(13.0)	11(26.2)	0.06	13(16.1)	11(18.0)	0.75	11.0(10.0–12.5)	0.46
No	6.0(6.0–8.0)		87(87.0)	31(73.8)		68(83.9)	50(81.2)		11.0(9.0–12.0)	
Previous chronic lung disease										
Yes	6.0(6.0–8.0)	0.53	40(40.0)	29(69.1)	**0.002**	37(45.7)	32(52.5)	0.42	11.0(9.0–12.0)	0.25
No	6.0(6.0–8.0)		60(60.0)	13(30.9)		44(54.3)	29(47.5)		11.0(10.0–13.0)	
Previous depression or anxiety										
Yes	6.0(6.0–7.0)	0.68	25(25.3)	20(47.6)	**0.009**	15(18.8)	30(49.2)	**0.0001**	11.0(9.0–12.0)	0.19
No	6.0(6.0–8.0)		74(74.7)	22(52.4)		65(81.2)	31(50.8)		11.0(9.0–13.0)	

Data are presented as median (IQR) or count (%).

The n column may not add up to 143 if missing characteristic data. Bold *p*-values represent statistically significant results.

* The lung cancer risk construct is missing data for five respondents.

† Health perceptions construct missing data for one respondent.

**Table 4.** Health beliefs and expectancies domain results.

	Self-efficacy n = 140*		Beliefs about LCS early detection n = 143		LCS knowledge n = 143^†^	

Cronbach alpha	0.88		0.72		0.70	
Median overall score (IQR)	35.0(32.0–36.0)		14.0(11.0–15.0)		4.0(2.0–6.0)	
Scale answer range	12.0–36.0		3.0–15.0		0.0–12.0	
Sex						
Male	35.0(32.0–36.0)	0.58	14.0(11.0–15.0)	0.92	3.0(2.0–5.0)	**<0.0001**
Female	35.0(32.0–36.0)		14.0(11.0–15.0)		6.0(4.0–7.5)	
Race/Ethnicity						
Non-Hispanic White	35.0(32.0–36.0)	0.92	14.0(11.0–15.0)	0.96	4.0(3.0–6.0)	**0.03**
Other race/ethnicity	35.0(33.0–36.0)		15.0(11.0–15.0)		3.0(2.0–4.0)	
Age						
50–64	35.0(32.0–36.0)	0.13	14.0(12.0–15.0)	0.13	5.0(3.0–6.0)	0.09
65–77	36.0(33.0–36.0)		14.0(11.0–15.0)		4.0(2.0–6.0)	
Smoking status						
Current	35.0(33.0–36.0)	0.60	13.0(11.5–15.0)	0.22	4.0(2.5–6.0)	0.48
Former	35.0(32.0–36.0)		14.0(11.0–15.0)		4.0(2.0–6.0)	
Insurance						
Private	35.0(33.0–36.0)	0.66	14.5(12.0–15.0)	0.28	5.0(3.0–6.0)	0.18
Government	35.0(31.0–36.0)		14.0(11.0–15.0)		4.0(2.0–6.0)	
Education						
High School	34.0(31.0–36.0)	0.53	13.0(10.0–15.0)	0.62	4.0(2.0–6.0)	0.39
College	35.0(32.0–36.0)		14.0(12.0–15.0)		4.0(2.0–6.0)	
Income						
<$75,000	34.0(30.0–36.0)	**0.03**	13.0(11.0–15.0)	0.12	4.0(2.0–6.0)	0.59
>$75,000	36.0(34.0–36.0)		15.0(12.0–15.0)		4.0(2.0–6.0)	
Family history of lung cancer						
Yes	35.0(31.5–36.0)	0.63	13.5(11.0–15.0)	0.62	4.0(2.5–6.0)	0.76
No	35.0(32.0–36.0)		14.0(11.0–15.0)		4.0(2.0–6.0)	
Previous cancer diagnosis						
Yes	35.0(33.0–36.0)	0.64	14.0(11.0–15.0)	0.93	4.0(2.0–6.0)	0.67
No	35.0(32.0–36.0)		14.0(11.0–15.0)		4.0(3.0–6.0)	
Previous diabetes diagnosis						
Yes	36.0(35.0–36.0)	**0.02**	13.5(12.0–15.0)	0.65	4.0(3.0–6.0)	0.88
No	35.0(32.0–36.0)		14.0(11.0–15.0)		4.0(2.0–6.0)	
Previous HBP diagnosis						
Yes	35.0(31.0–36.0)	0.57	14.0(11.0–15.0)	0.08	4.0(2.0–6.0)	0.21
No	35.0(33.0–36.0)		14.0(13.0–15.0)		4.0(3.0–6.0)	
Previous heart condition diagnosis						
Yes	36.0(35.0–36.0)	**0.03**	13.0(11.0–15.0)	0.45	4.0(2.0–5.5)	0.32
No	35.0(32.0–36.0)		14.0(11.0–15.0)		4.0(3.0–6.0)	
Previous chronic lung disease						
Yes	35.0(30.5–36.0)	0.72	13.0(11.0–15.0)	0.10	4.0(3.0–6.0)	0.34
No	35.0(32.0–36.0)		14.0(12.0–15.0)		4.0(2.0–6.0)	
Previous depression or anxiety						
Yes	31.5(32.0–36.0)	0.40	14.0(11.0–15.0)	0.83	3.0(5.0–7.0)	0.07
No	35.0(32.0–36.0)		14.0(11.0–15.0)		4.0(2.0–6.0)	

Data are presented as median (IQR).

Bold p-values represent statistically significant results.

* The self-efficacy construct is missing data for three respondents.

† Knowledge questions with missing answers were recoded to ‘No’.

**Table 5.** Affects (emotions) domain results.

	Stigma n = 139*	p-value	Fatalism n = 143^†^	p-value	Worry about diagnosis n = 143	p-value

Cronbach alpha	0.67		0.82		0.91	
Median overall score (IQR)	14.0(12.0–14.0)		1.0(0.0–2.0)		11.0(9.0–12.0)	
Scale answer range	5.0–19.0		0.0–11.0		3.0–15.0	
Sex						
Male	13.0(12.0–14.0)	0.18	1.0(0.0–2.0)	0.37	11.0(9.0–12.0)	0.45
Female	14.0(13.0–14.0)		1.0(0.0–2.0)		12.0(9.0–13.0)	
Race/Ethnicity						
Non-Hispanic White	14.0(12.0–14.0)	0.94	1.0(0.0–2.0)	0.47	12.0(9.0–13.0)	0.31
Other race/ethnicity	13.5(11.5–15.5)		1.0(0.0–4.0)		10.0(7.0–12.0)	
Age						
50–64	13.0(13.0–14.0)	0.82	1.0(0.0–2.0)	0.60	12.0(10.0–13.0)	**0.01**
65–77	14.0(12.0–14.0)		1.0(0.0–3.0)		10.0(8.0–12.0)	
Smoking status						
Current	13.0(12.0–14.0)	0.35	1.0(0.0–2.5)	0.52	11.0(9.0–13.0)	0.77
Former	14.0(12.0–14.0)		1.0(0.0–2.0)		12.0(9.0–12.0)	
Insurance						
Private	14.0(13.0–14.0)	0.43	1.0(0.0–2.0)	0.93	10.5(9.0–12.0)	0.68
Government	14.0(12.0–14.0)		1.0(0.0–2.0)		12.0(9.0–13.0)	
Education						
High School	12.5(11.5–14.0)	**0.02**	1.0(1.0–1.5)	**0.01**	10.0(8.0–13.0)	0.64
College	14.0(13.0–14.0)		1.0(0.0–2.0)		12.0(9.0–12.0)	
Income						
<$75,000	13.0(12.0–14.0)	0.35	1.0(0.0–2.0)	0.61	11.0(9.0–12.0)	0.94
>$75,000	14.0(13.0–14.0)		1.0(0.0–1.0)		12.0(9.0–13.0)	
Family history of lung cancer						
Yes	14.0(12.0–14.0)	0.90	1.0(1.0–3.5)	**0.01**	12.0(10.0–13.0)	**0.05**
No	13.0(12.0–14.0)		1.0(0.0–2.0)		11.0(9.0–12.0)	
Previous cancer diagnosis						
Yes	14.0(13.0–14.0)	0.69	1.0(0.0–1.0)	0.18	11.0(10.0–13.0)	0.35
No	13.5(12.0–14.0)		1.0(0.0–2.0)		11.5(9.0–12.0)	
Previous diabetes diagnosis						
Yes	13.0(12.0–14.0)	0.83	1.0(0.0–1.0)	0.14	12.0(9.0–12.0)	0.88
No	14.0(12.0–14.0)		1.0(0.0–2.0)		11.0(9.0–12.0)	
Previous HBP diagnosis						
Yes	13.0(12.0–14.0)	0.57	1.0(0.0–2.0)	0.18	11.0(9.0–12.0)	0.32
No	14.0(13.0–14.0)		1.0(0.0–1.0)		12.0(9.0–13.0)	
Previous heart condition diagnosis						
Yes	13.0(12.5–14.0)	0.76	1.0(0.0–2.5)	0.86	9.5(8.0–12.0)	0.22
No	14.0(12.0–14.0)		1.0(0.0–2.0)		12.0(9.0–13.0)	
Previous chronic lung disease						
Yes	14.0(12.0–14.0)	0.46	1.0(0.0–2.0)	0.91	12.0(9.0–13.0)	0.19
No	13.0(12.0–14.0)		1.0(0.0–2.0)		10.0(9.0–12.0)	
Previous depression or anxiety						
Yes	13.0(12.0–14.0)	0.54	1.0(0.0–1.0)	0.20	12.0(10.0–14.0)	**0.006**
No	14.0(12.5–14.0)		1.0(0.0–2.5)		10.0(9.0–12.0)	

Data are presented as median (IQR).

Bold *p*-values represent statistically significant results.

* The stigma construct is missing data for four respondents.

† Fatalism questions with missing answers were recoded to ‘No’.

**Table 6.** Health goals and values domain results.

	Perceived benefits n = 138*	*p*-value	Belief in a healthy lifestylen = 138*	*p*-value	Illness coherence n = 140^†^	*p*-value	Consequence of diagnosis n = 142^‡^	*p*-value

Cronbach alpha	0.86		0.84		0.74		0.88	
Median overall score (IQR)	22.0(18.0–24.0)		37.4(±6.7)		8.0(6.0–10.0)		14.0(12.0–15.0)	
Range of answers	14.0–24.0		21.0–52.0		3.0–15.0		3.0–15.0	
Sex								
Male	22.0(18.0–24.0)	0.91	37.8(±6.6)	0.49	8.0(6.0–10.0)	0.23	14.0(12.0–15.0)	0.96
Female	22.0(18.0–24.0)		37.0(±6.9)		9.0(6.0–11.0)		14.0(12.0–15.0)	
Race/Ethnicity								
Non-Hispanic White	21.0(18.0–24.0)	0.16	37.5(±6.4)	0.98	8.5(6.0–10.0)	0.18	14.0(12.0–15.0)	0.45
Other Race/Ethnicity	24.0(20.0–24.0)		37.5(±9.1)		7.0(5.0–10.0)		14.0(14.0–15.0)	
Age								
50–64	21.5(18.0–24.0)	0.93	37.1(±6.4)	0.61	9.0(6.0–11.0)	0.49	14.0(13.0–15.0)	0.38
65–77	22.0(18.0–24.0)		37.8(±7.1)		8.0(6.0–11.0)		14.0(12.0–15.0)	
Smoking status								
Current	21.0(18.0–24.0)	0.33	36.0(±7.3)	0.09	8.0(6.0–11.0)	0.65	14.5(12.0–15.0)	0.83
Former	22.0(19.0–24.0)		38.1(±6.4)		8.0(6.0–10.0)		14.0(13.0–15.0)	
Insurance								
Private	22.0(18.0–24.0)	0.61	37.5(±6.4)	0.90	8.5(6.0–11.0)	0.81	15.0(13.0–15.0)	**0.05**
Government	22.0(18.0–24.0)		37.4(±6.9)		8.0(6.0–10.0)		14.0(12.0–15.0)	
Education								
High School	20.0(18.0–23.0)	0.10	34.5(±5.6)	**0.01**	7.0(6.0–10.0)	0.09	13.0(12.0–15.0)	0.13
College	22.0(19.0–24.0)		38.1(±6.8)		9.0(6.0–10.0)		14.0(12.0–15.0)	
Income								
<$75,000	22.0(18.0–24.0)	0.87	36.6(±7.0)	0.17	8.0(6.0–10.0)	0.94	14.0(12.0–15.0)	0.15
>$75,000	22.0(19.0–24.0)		38.0(±6.2)		9.0(6.0–10.5)		15.0(12.0–15.0)	
Family history of lung cancer								
Yes	20.0(18.0–24.0)	0.51	36.5(±7.0)	0.39	9.0(6.0–11.0)	0.35	15.0(13.0–15.0)	0.19
No	22.0(19.0–24.0)		37.7(±6.7)		8.0(6.0–10.0)		14.0(12.0–15.0)	
Previous cancer diagnosis								
Yes	21.5(19.0–24.0)	0.69	39.2(±5.4)	0.11	8.5(6.0–11.5)	0.85	15.0(13.0–15.0)	0.19
No	22.0(18.0–24.0)		36.9(±7.0)		8.0(6.0–10.0)		14.0(12.0–15.0)	
Previous diabetes diagnosis								
Yes	21.0(18.0–24.0)	0.84	37.3(±6.1)	0.92	8.0(6.0–10.0)	0.45	14.0(13.0–15.0)	0.61
No	22.0(18.0–24.0)		37.4(±7.0)		8.0(6.0–11.0)		14.0(12.0–15.0)	
Previous HBP diagnosis								
Yes	21.5(18.0–24.0)	0.51	37.0(±6.3)	0.41	8.0(6.0–10.0)	**0.03**	14.0(12.0–15.0)	0.76
No	22.0(20.0–24.0)		38.0(±7.4)		9.0(7.0–11.0)		14.0(12.0–15.0)	
Previous heart condition diagnosis								
Yes	18.0(19.0–23.0)	0.11	38.3(±6.1)	0.42	8.0(6.0–10.0)	0.40	13.5(12.0–15.0)	0.11
No	22.0(19.0–24.0)		37.1(±6.8)		8.0(6.0–10.0)		13.0(12.0–15.0)	
Previous chronic lung disease								
Yes	21.0(18.0–24.0)	0.33	36.7(±6.6)	0.25	9.0(7.0–10.0)	0.27	14.0(12.5–15.0)	0.18
No	22.0(18.0–24.0)		38.1(±6.8)		8.0(6.0–10.0)		15.0(12.0–15.0)	
Previous depression or anxiety								
Yes	22.0(18.0–24.0)	0.75	36.5(±6.0)	0.31	8.0(6.0–10.0)	0.84	14.0(12.0–15.0)	0.92
No	22.0(18.0–24.0)		37.7(±6.9)		8.0(6.0–10.0)		14.0(12.0–15.0)	

Data are presented as median (IQR) or mean (±standard deviation).

Bold *p*-values represent statistically significant results.

* Perceived benefits and healthy lifestyle constructs are missing data for five respondents.

† The illness coherence construct is missing data for three respondents.

‡ The consequence of the diagnosis construct is missing data for one respondent.

**Table 7.** Self-regulatory competencies domain results.

	Perceived barriers n = 138*	*p*-Value	Personal control n = 139^‡^	*P*-Value	Treatment control n = 140∞	*p*-Value	Health literacy n = 140∞	*p*-Value	Numeracy n = 138*	*p*-Value	Medical minimizer maximizer n = 142^†^		*p*-Value

Cronbach alpha	0.94		0.77		0.70		0.73		0.91		NA		NA
Median overall score (IQR)	24.5 (17.0–32.0)		12.0 (11.0–13.0)		10.0 (9.0–12.0)		18.0 (15.0–20.0)		38.5 (30.0–44.0)		Minimizer n = 76	Maximizer n = 66	
Range of answers	17.0–46.0		3.0–15.0		3.0–15.0		5.0–20.0		8.0–48.0				
Sex													
Male	26.0 (18.0–34.0)	0.31	12.0 (11.0–13.0)	0.83	10.0 (9.5–12.0)	0.36	18.0 (15.0–20.0)	0.27	40.0 (34.0–45.0)	0.16	43 (56.6)	38 (58.5)	0.23
Female	24.0 (17.0–31.0)		12.0 (10.0–14.0)		10.0 (9.0–12.0)		18.0 (15.0–20.0)		37.0 (29.0–43.0)		33 (43.4)	27 (41.5)	
Race/Ethnicity													
Non-Hispanic White	25.0 (17.0–32.0)	0.40	12.0 (10.0–13.0)	0.72	10.0 (9.0–12.0)	**0.03**	18.0 (15.0–20.0)	0.86	39.0 (33.0–44.0)	0.10	66 (90.4)	58 (90.6)	0.97
Other race/ethnicity	18.5 (17.0–31.0)		12.0 (11.0–14.0)		11.0 (11.0–14.0)		18.0 (15.0–20.0)		30.0 (17.0–45.0)		7 (9.6)	6 (9.4)	
Age													
50–64	25.0 (19.0–30.0)	0.67	12.0 (11.0–14.0)	**0.04**	11.0 (10.0–12.0)	0.36	18.0 (14.0–20.0)	0.29	39.0 (33.0–44.5)	0.97	27 (37.0)	31 (48.4)	0.18
65–77	23.5 (17.0–33.0)		12.0 (10.0–13.0)		10.0 (9.0–12.0)		18.0 (16.0–20.0)		39.0 (30.0–44.0)		46 (63.0)	33 (51.6)	
Smoking status													
Current	22.5 (17.0–30.0)	0.21	12.0 (11.0–14.0)	0.74	10.0 (9.0–12.0)	0.63	18.0 (15.0–20.0)	0.45	38.0 (29.0–45.0)	0.67	23 (30.7)	21 (31.8)	0.88
Former	25.0 (18.0–33.0)		12.0 (11.0–13.0)		10.0 (9.0–12.0)		18.0 (15.0–20.0)		39.0 (32.0–43.0)		52 (69.3)	45 (68.2)	
Insurance													
Private	20.5 (18.0–28.0)	0.61	12.0 (11.0–14.0)	0.45	10.0 (10.0–12.0)	0.79	19.0 (15.5–20.0)	0.19	40.0 (34.0–46.0)	0.13	22 (29.0)	22 (33.8)	0.53
Government	26.0 (17.0–34.0)		12.0 (10.0–13.0)		10.0 (9.0–12.0)		18.0 (15.0–20.0)		38.0 (29.5–43.0)		54 (71.0)	43 (66.2)	
Education													
High School	25.0 (17.0–33.0)	0.95	11.5 (10.0–13.0)	0.13	10.0 (9.0–12.0)	0.68	15.0 (14.0–18.0)	**0.001**	27.0 (22.0–33.0)	**<0.0001**	19 (25.3)	7 (10.9)	**0.03**
College	25.0 (18.0–32.0)		12.0 (11.0–13.0)		10.0 (9.0–12.0)		19.0 (15.0–20.0)		40.0 (35.0–45.0)		56 (74.7)	56 (89.1)	
Income													
< $75,000	27.5 (17.5–34.0)	0.12	12.0 (11.0–13.0)	0.66	10.0 (10.0–12.0)	0.75	16.0 (14.0–19.0)	**0.002**	35.0 (27.0–41.0)	**<0.0001**	37 (52.1)	32 (50.8)	0.88
> $75,000	21.5 (18.0–30.0)		12.0 (10.0–13.0)		10.0 (9.0–12.0)		19.0 (17.0–20.0)		41.0 (37.0–46.0)		34 (47.9)	31 (49.2)	
Family history of lung cancer													
Yes	19.0 (17.0–34.0)	0.13	12.0 (11.0–14.0)	0.60	10.0 (9.0–11.0)	**0.01**	18.0 (15.0–20.0)	0.54	37.5 (29.0–44.0)	1.00	23 (30.3)	13 (19.7)	0.15
No	26.0 (18.0–33.0)		12.0 (11.0–13.0)		11.0 (10.0–12.0)		18.0 (15.0–20.0)		39.0 (30.5–43.0)		53 (69.7)	53 (80.3)	
Previous cancer diagnosis													
Yes	23.5 (18.0–34.0)	0.78	12.0 (11.0–13.0)	0.88	11.0 (9.0–12.0)	0.41	17.0 (15.0–20.0)	0.80	40.0 (34.0–43.0)	0.41	13 (17.1)	16 (25.0)	0.25
No	24.0 (17.0–32.0)		12.0 (11.0–13.0)		10.0 (9.0–12.0)		18.0 (15.0–20.0)		38.0 (30.0–44.0)		63 (82.9)	48 (75.0)	
Previous diabetes diagnosis													
Yes	19.0 (17.0–32.0)	0.29	12.0 (10.0–13.0)	0.15	10.0 (9.0–12.0)	0.76	18.0 (15.0–20.0)	0.61	39.0 (27.0–43.0)	0.28	18 (23.7)	20 (30.8)	0.34
No	25.5 (18.0–32.0)		12.0 (11.0–14.0)		10.0 (9.0–12.0)		18.0 (15.0–20.0)		38.5 (32.0–44.0)		58 (76.3)	45 (69.2)	
Previous HBP diagnosis													
Yes	26.0 (17.0–34.0)	0.25	12.0 (10.0–13.0)	0.27	10.0 (9.0–12.0)	0.80	18.0 (15.0–20.0)	0.87	38.0 (29.0–42.0)	0.15	46 (60.5)	38 (57.6)	0.72
No	23.0 (18.0–28.0)		12.0 (11.0–14.0)		10.0 (9.0–12.0)		18.0 (15.0–20.0)		40.0 (33.0–45.0)		30 (39.5)	28 (42.4)	
Previous heart condition diagnosis													
Yes	28.0 (18.0–34.0)	0.18	12.0 (9.5–13.0)	0.88	10.0 (10.0–12.0)	0.83	15.0 (14.0–17.0)	**0.0003**	37.5 (28.0–40.0)	**0.03**	13 (17.1)	11 (16.9)	0.98
No	24.0 (17.0–31.0)		12.0 (11.0–13.0)		10.0 (9.0–12.0)		18.0 (16.0–20.0)		40.0 (33.0–45.0)		63 (89.9)	54 (83.1)	
Previous chronic lung disease													
Yes	28.0 (19.0–34.0)	**0.005**	12.0 (10.0–13.0)	0.56	10.0 (9.0–11.0)	0.21	18.0 (15.0–20.0)	0.44	37.5 (29.5–43.0)	0.16	36 (47.4)	33 (50.8)	0.64
No	20.0 (17.0–28.0)		12.0 (11.0–14.0)		10.5 (9.0–12.0)		18.0 (15.0–20.0)		40.0 (33.0–45.0)		40 (52.6)	32 (49.2)	
Previous depression or anxiety													
Yes	24.0 (17.0–32.0)	0.84	12.0 (10.0–13.5)	0.61	10.0 (9.0–11.0)	0.25	18.0 (14.5–20.0)	0.34	40.0 (31.5–44.0)	0.92	18 (23.7)	27 (42.2)	**0.02**
No	25.0 (18.0–32.0)		12.0 (11.0–13.0)		10.0 (9.0–12.0)		18.0 (15.0–20.0)		38.5 (31.5–44.0)		58 (76.3)	37 (57.8)	

Data are presented as median (IQR) or n (%).

Note that the n column may not add up to 143 if missing characteristic data.

Bold *p*-values represent statistically significant results.

* Perceived barriers and numeracy constructs are missing data for five respondents.

† The medical minimizer/maximizer construct is missing data for one respondent.

‡ Personal control construct missing data for four respondents.

∞ Treatment control and literacy constructs are missing data for three respondents.

**Table 8.** Possible options for communication/intervention targeting or tailoring.

	Age	Sex	Race/ ethnicity	Smoking status	Insurance	Education	Income	Family history	Previous cancer	Previous diabetes	Previous hbp	Previous heart condition	Previous chronic lung	Previous anxiety/depression

**Health relevant encodings**														
Perceived lung cancer risk				X				X						
Self-health rating					X	X	X						X	X
Ability to take care of health		X		X										X
**Health beliefs and expectancies**														
LCS self-efficacy							X			X		X		
Early detection beliefs														
Knowledge		X	X											
**Affects (emotions)**														
Stigma						X								
Fatalism						X		X						
Diagnosis worry	X							X						X
**Health goals and values**														
Perceived benefits														
Healthy lifestyle						X								
Illness coherence											X			
Diagnosis consequences					X									
**Self-regulatory competencies**														
Perceived barriers													X	
Personal control	X													
Treatment control			X											
Literacy						X	X					X		
Numeracy						X	X					X		
Medical minimizer/maximizer						X								

An ‘X’ represents each statistically significant difference between survey measures and respondent attributes.

## Data Availability

Data are available on request from the corresponding author.
