# Supplementary material for: Describing behavioral aspects of annual lung cancer screening participation: Application of the cognitive-social health information processing model
Source: Prev Oncol Epidemiol. Author manuscript; Available in PMC 2026 Aug 29. (PMC13524418; doi:10.1080/28322134.2026.2690703)
Supplement: Supplementary file 1 [file NIHMS2197337-supplement-Supp_1.docx]

| **Survey Section** | **Relevant LCS Construct** | **Measure/ scale** |
| --- | --- | --- |
| 1) Health-relevant encodings   (C-SHIP domain) | Lung cancer risk perception | LCS Health Belief Scales – Perceived Risks Scale (Carter-Harris et al., 2017) |
|  | Functional well-being/health | In general, how would you rate your health? - Adapted from HINTS cycle 5 (National Cancer Institute, 2020) |
|  | Co-morbidities/ life expectancy | Health Perceptions Questionnaire–Health worry/concern scale (Ware, 1976) |
| 2) Health beliefs and expectancies (C-SHIP domain) | Self-efficacy | LCS Health Belief Scales – Self-Efficacy Scale (Carter-Harris et al., 2017) |
|  | Beliefs about LCS for early detection | Self-Regulatory Questionnaire for Lung Cancer Screening – Behavioral response subscale (Quaife et al., 2022) |
|  | LCS specific knowledge | LCS Knowledge Survey (Volk et al., 2014) |
| 3) Affects (emotions) (C-SHIP domain) | Lung Cancer Stigma | Cataldo Lung Cancer Stigma Scale – Smoking subscale (Cataldo et al., 2011) |
|  | Fatalism | Revised Powe Fatalism Inventory (Mayo et al., 2001) |
|  | Worry about cancer diagnosis | Self-Regulatory Questionnaire for Lung Cancer Screening- emotional representation scale (Quaife et al., 2022) |
| 4) Health goals and values (C-SHIP domain) | LCS risks and benefits | LCS Health Belief Scales – Perceived Risk & Benefit Scales (Carter-Harris et al., 2017) |
|  | Overall belief in healthy lifestyle | Health Behavior Scale for Cancer Patients (Cecon et al., 2021) |
|  | Importance or early detection | Self-Regulatory Questionnaire for Lung Cancer Screening – Illness coherence subscale (Quaife et al., 2022) |
|  | Consequence of a lung cancer diagnosis | Self-Regulatory Questionnaire for Lung Cancer Screening – Consequences subscale (Quaife et al., 2022) |
|  | Barriers to continued screening | LCS Health Belief Scales – Perceived Barriers Scale (Carter-Harris et al., 2017) |
| 5) Self-regulatory competencies (C-SHIP domain) | Health info seeking behavior | Medical Minimizer Maximizer Scale 1 (MMS-1) (Scherer and Zikmund-Fisher, 2020) |
|  | Control over cancer diagnosis | Self-Regulatory Questionnaire for Lung Cancer Screening – Personal control and treatment control subscales (Quaife et al., 2022) |
|  | Health literacy (subjective) | BRIEF Health Literacy Scale (Haun et al., 2012) |
|  | Health numeracy (subjective) | Subjective Numeracy Scale (Fagerlin et al., 2007) |

**Supplemental Table 1: Quantitative survey content.** Application of lung cancer screening constructs and validated measures as operationalized by the Cognitive Social Health Information Processing model.

| Knowledge question (correct answer)* | Answered correctly  n (%) | Answered incorrectly  n (%) |
| --- | --- | --- |
| 1) Is lung cancer the second leading cause of cancer death in the US? (No) | 6 (4.2) | 137 (95.8) |
| 2) Is smoking responsible for more than half of all lung cancer deaths in the US? (Yes) | 87 (60.4) | 56 (39.2) |
| 3) Does having a low dose CT decrease your chances of getting lung cancer? (No) | 101 (70.6) | 42 (29.4) |
| 4) Does having a low dose CT decrease your chances of dying from lung cancer? (Yes) | 66 (46.2) | 77 (53.8) |
| 5) If nothing is found on your low dose CT scan should you repeat the scan in 6 months? (No) | 71 (49.7) | 72 (50.3) |
| 6) Is radiation exposure one of the potential harms of screening? (Yes) | 49 (34.3) | 94 (65.7) |
| 7) Can a low dose CT show that you have a tumor when you do not? (Yes) | 20 (14.0) | 123 (86.0) |
| 8) Can a low dose CT miss a tumor in your lungs? (Yes) | 49 (34.3) | 94 (65.7) |
| 9) Will all tumors found in the lungs grow to be life threatening? (No) | 71 (49.7) | 72 (50.3) |
| 10) Should all individuals that currently and formerly smoke cigarettes be screened for lung cancer? (No) | 4 (2.8) | 139 (97.2) |
| 11) Can a low dose CT find heart disease? (Yes) | 37 (25.9) | 106 (74.1) |
| 12) Can a low dose CT lower your chances of dying from lung cancer by 40%? (Yes) | 31 (21.7) | 112 (78.3) |
| 13) Will most people with suspicious cancer results actually be diagnosed with lung cancer? (No) | 25 (17.5) | 118 (82.5) |
| 14) Will about a quarter of people screened with low dose CT have a results that is suspicious for lung cancer? (Yes) | 15 (10.5) | 128 (89.5) |

**Supplemental Table 2. Responses for individual knowledge questions**

*Questions are from Volk, 2014.
